# Effect of aminergic signaling on the humoral innate immunity response of *Drosophila*

**DOI:** 10.3389/fphys.2023.1249205

**Published:** 2023-08-24

**Authors:** Giulia Cattabriga, Giorgia Giordani, Giuseppe Gargiulo, Valeria Cavaliere

**Affiliations:** ^**1**^ Dipartimento di Farmacia e Biotecnologie, Alma Mater Studiorum Università di Bologna, Bologna, Italy; ^**2**^ Interuniversity Center for Studies on Bioinspired Agro-Environmental Technology (BAT Center), University of Napoli “Federico II”, Naples, Italy

**Keywords:** dopamine, serotonin, octopamine, neuroimmunity, drosomycin, neurogenetic manipulation, aminergic signaling downregulation

## Abstract

Biogenic amines are crucial signaling molecules that modulate various physiological life functions both in vertebrates and invertebrates. In humans, these neurotransmitters influence the innate and adaptive immunity systems. In this work, we analyzed whether the aminergic neurotransmission of dopamine, serotonin, and octopamine could have an impact on the humoral innate immune response of *Drosophila melanogaster*. This is a powerful model system widely used to uncover the insect innate immunity mechanisms which are also conserved in mammals. We found that the neurotransmission of all these amines positively modulates the Toll-responsive antimicrobial peptide (AMP) *drosomycin* (*drs*) gene in adult flies infected with the *Micrococcus luteus* bacterium. Indeed, we showed that either blocking the neurotransmission in their specific aminergic neurons by expressing shibire^ts^ (Shi^ts^) or silencing the vesicular monoamine transporter gene (*dVMAT*) by RNAi caused a significantly reduced expression of the Toll-responsive *drs* gene. However, upon *M. luteus* infection, the block of aminergic transmission did not alter the expression of AMP *attacin* genes responding to the immune deficiency (Imd) and Toll pathways. Overall, our results not only reveal a neuroimmune function for biogenic amines in humoral immunity but also further highlight the complexity of the network controlling AMP gene regulation.

## 1 Introduction

The fruit fly *Drosophila melanogaster* has been widely used as a model system to study the insect innate immunity, and it has led to relevant breakthroughs in the immunity field (Lemaitre and Hoffmann, 2007). In *Drosophila*, humoral antimicrobial peptides (AMPs) and the cellular response by hemocytes are the two major reactions that fight pathogens. AMPs are produced in response to microbial infection, either locally at various surface epithelia or systemically by the fat body (the equivalent of the mammalian liver) which secretes AMPs into hemolymph (Imler and Bulet, 2005). Two NF-κB pathways, Toll and immune deficiency (Imd) pathways, regulate the transcription of AMP genes. The Toll pathway is mostly activated by Gram-positive bacteria and fungi, while the Imd pathway largely responds to Gram-negative bacteria (Lemaitre and Hoffmann, 2007). Some AMP genes specifically respond to one of these pathways, while others appear under the control of both pathways (De Gregorio et al., 2002; Tanji et al., 2007).

The insect antimicrobial response evidences numerous similarities to the innate immune response in mammals (Hargreaves and Medzhitov, 2005), and many components of the innate immune system have been conserved during metazoan evolution. A growing body of data produced over the years clearly indicates the occurrence of an intense and very complex neuro-immune cross talk, which modulates the defense responses in humans (Pavlov et al., 2018; Hodo et al., 2020; Tarnawski and Olofsson, 2021). In insects, these neuroimmune interactions are very poorly studied, although a more holistic vision of immune homeostasis has been developed, trying to understand how the immune response is regulated at the organism level by the microbiota, metabolism, and endocrine system (Buchon et al., 2014). Recently, we contributed to this topic and showed the role of neurotransmitter acetylcholine in modulating the humoral innate immunity of *Drosophila* (Giordani et al., 2023). In humans, it has been clearly demonstrated that acetylcholine signaling is involved in maintaining immunological homeostasis (Andersson and Tracey, 2012). In the neuroimmune cross talk, aminergic signaling also plays an important role in regulating innate and adaptive immunity systems (Hodo et al., 2020). In the central nervous system of both vertebrates and invertebrates, biogenic amines are important signaling molecules that control and regulate many vital functions. In *Drosophila*, these neuroactive molecules take part in the regulation of various physiological processes, such as locomotion, sexual behavior, feeding, sleep/arousal, learning/memory, and many other homeostatic mechanisms (Monastirioti, 1999; Blenau and Baumann, 2001).

Here, we investigated the neuroimmune activity of *Drosophila* biogenic amines by focusing on dopamine, serotonin, and octopamine signaling. Dopamine and serotonin are found in both invertebrates and mammals, while octopamine is the invertebrate analog of norepinephrine.

We analyzed the effect on the expression of the AMP *drosomycin* (*drs*) as a readout of the Toll pathway upon immune challenge in genetic backgrounds impairing specific aminergic signaling in the nervous system. Interestingly, blocking dopaminergic, serotonergic, or octopaminergic neurotransmission caused a reduced level of *drs* expression.

The finding that dopamine, serotonin, and octopamine signaling acts positively on the *drs* expression, which responds to the Toll pathway, strengthens the previously described interaction between the nervous system and humoral innate immunity of *Drosophila* (Giordani et al., 2023). However, from our presented data, it appears that the aminergic signaling of dopamine, serotonin, and octopamine does not target the Imd signaling that activates the expression of AMP *attacin* genes.

## 2 Materials and methods

### 2.1 *Drosophila* stocks and crosses

*Drosophila* stocks were maintained on standard cornmeal/yeast medium at 25°C under a 12:12 h light/dark cycle. The stocks *Th*-*GAL4* (#8848), *Tdc2*-*GAL4* (#9313), *dVMAT*-*RNAi* (#44471), *w* ^*1118*^ (#5905), and *attP2* control line for TRiP RNAi lines (#36303) were obtained from the Bloomington *Drosophila* Stock Center. The *UAS*-*shi* ^*ts*^ and *Tph*-*GAL4* lines were received as kind gifts from James Jepson and Laurent Saugnet, respectively.

For the *shibire* ^*ts*^ experiments, *UAS*-*shi* ^*ts*^ female flies were crossed with male flies of the different aminergic GAL4 driver lines at 21°C. For the *dVMAT-RNAi* experiments, *UAS*-*dVMAT*-*RNAi* female flies were crossed with male flies of the different aminergic GAL4 driver lines at 25°C. The proper control flies were obtained by crossing (at the same temperature used for the relative aforementioned crosses) the GAL4 lines with the strains in which the UAS construct insertions were generated. For *shi* ^*ts*^, we used the *w* ^*1118*^ strain, while for *dVMAT*-*RNAi*, we used the *attP2* strain.

### 2.2 Immune challenges of adult flies

One-day-old adult female flies were collected and put on fresh food. For the *dVMAT*-*RNAi* experiments, the flies were maintained at 25°C for 1 day and shifted to 29°C for 2 more days, while for the *shi* ^*ts*^ experiments, the flies were maintained for 3 more days at 21°C and shifted to 29°C for 1 h before the immune challenge. The Gram-positive *Micrococcus luteus* bacterium (a kind gift from Bruno Lemaitre) was precultured in the LB medium. To introduce the bacteria into the body cavity, pre-anesthetized female flies were pricked into the lateral side of the thorax using a sharpened needle dipped in a concentrated *M. luteus* suspension (OD_600_ = 500) and transferred to new vials with fresh food. The flies were incubated at 29°C and sampled for analysis for 6 h after infection.

### 2.3 qRT-PCR analysis

RNA extraction, reverse transcription, and qRT-PCR were performed, as previously described (Giordani et al., 2023). Total RNA was isolated from five adult female flies and quantified using the BioPhotometer^®^ (Eppendorf AG, Hamburg, Germany). A measure of 350 ng of total RNA was reverse-transcribed using the HiScript III^®^ RT SuperMix for qPCR (+gDNA wiper) kit (Vazyme Biotech, Nanjing, China). Expression levels of the genes were analyzed by qRT-PCR using SsoAdvanced Universal SYBR^®^ Green Supermix (Bio-Rad, Hercules, CA, United States) in the CFX Connect Real-Time PCR Detection System (Bio-Rad) through Bio-Rad Manager™ Software Version 3.1. *Rpl32* was used to normalize all data. The calibrator is given by a biological replicate of the not challenged (NC) control. Four biological replicates were used for each experiment. Primers used for qPCR were as follows:

*Rpl32* forward: 5′-GAC​GCT​TCA​AGG​GAC​AGT​ATC​TG-3′;

*Rpl32* reverse: 5′-AAA​CGC​GGT​TCT​GCA​TGA​G-3′.

*drs* forward: 5′-CGT​GAG​AAC​CTT​TTC​CAA​TAT​GAT​G-3′;

*drs* reverse: 5′-TCC​CAG​GAC​CAC​CAG​CAT-3′.

*attacin A* and *B* forward: 5′-CAC​AAC​TGG​CGG​AAC​TTT​GG-3′;

*attacin A* and *B* reverse: 5′-AAA​CAT​CCT​TCA​CTC​CGG​GC-3′.

*dVMAT* forward: 5′-GCC​CAT​CAT​ACC​CGA​GTT​CC-3′;

*dVMAT* reverse: 5′-GTT​GGA​CGT​TAA​AGG​TGT​GCG-3′.

### 2.4 Statistical analyses

Statistical analyses of qRT-PCR results were performed using GraphPad Prism version 8 (GraphPad software; San Diego, California, United States). All results are expressed as mean ± standard deviation (SD). Normality of the data was checked using the Shapiro–Wilk test, while homoscedasticity was checked using the F-test. A two-tailed unpaired *t*-test was used to compare the differences in the relative gene expression between the experimental and control groups at the same time point. In each graph, the relative *p*-value is reported for significant differences. Not significant (n.s.) = p≥ 0.05.

## 3 Results

### 3.1 Dopamine, serotonin, and octopamine positively regulate the *drosomycin* gene upon septic injury

**FIGURE 1:**
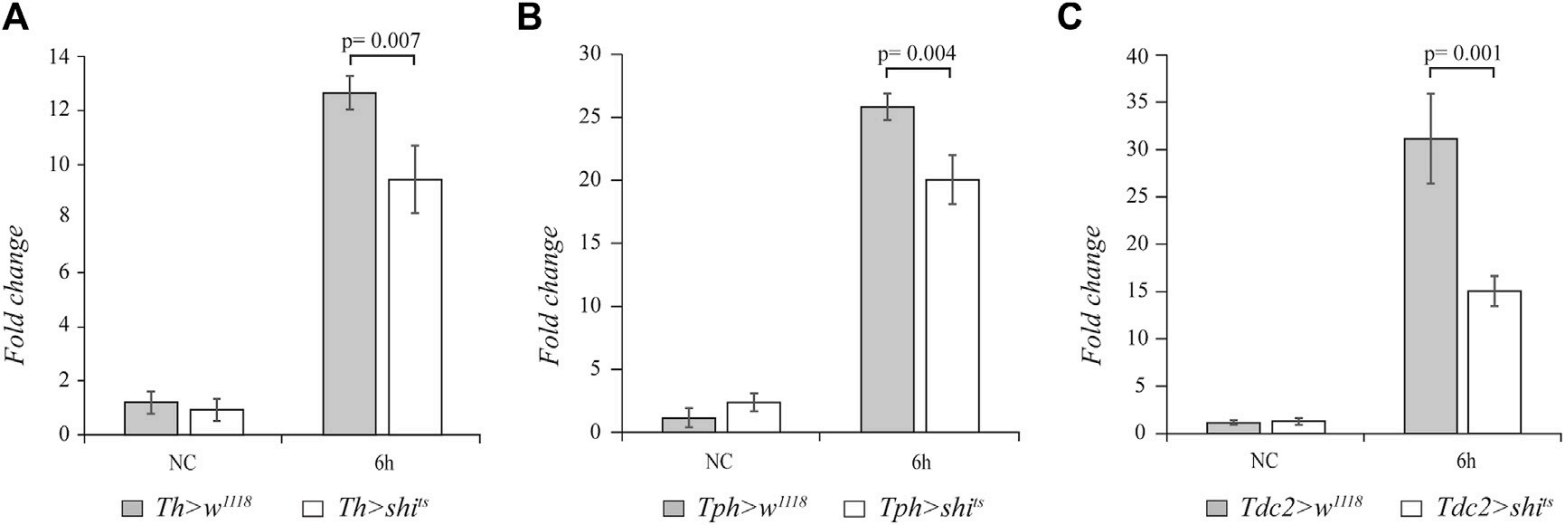
The block of aminergic neurotransmission alters *drs* expression. qRT-PCR analysis of *drs* transcript levels at 6 h after *Micrococcus luteus* infection of adult flies expressing the temperature-sensitive mutant of *shibire* gene (*shi* ^*ts*^) in dopaminergic **(A)**, serotonergic **(B)**, and octopaminergic neurons **(C)** compared to their controls.

In order to analyze whether the biogenic amines dopamine, serotonin, and octopamine are involved in regulating the systemic innate immune response of adult flies, we blocked the neurotransmission in the aminergic neurons. After the immune challenge with the Gram-positive *M. luteus* bacterium, we analyzed the expression of *drs* gene as a readout of the Toll pathway. To block dopamine neurotransmission, we used the temperature-sensitive mutant of *shibire* gene (*shi* ^*ts*^), the homologous of the mammalian *dynamin*, under the control of UAS sequences (*UAS*-*shi* ^*ts*^) (Kitamoto et al., 1998). The expression of this mutant at a restrictive temperature (above 29°C) inhibits vesicle recycling and synaptic transmission. We used the *tyrosine hydroxylase* (*Th*)-*GAL4* (Friggi-Grelin et al., 2003), *tryptophan hydroxylase* (*Tph*)-*GAL4* (Park et al., 2006), and *tyrosine decarboxylase* (*Tdc2*)-*GAL4* (Cole et al., 2005) drivers to specifically target the expression of *shi* ^*ts*^ in dopaminergic, serotonergic, and octopaminergic neurons, respectively. Before septic injury, 3–4-day-old female flies were shifted from 21°C to the restrictive temperature of 29°C for 1 h, and after infection, the flies were maintained at 29°C. We analyzed the effect on *drs* expression at 6 h after the immune challenge. Interestingly, a statistically significant decrease in the *drs* transcript level was caused by expressing *shi* ^*ts*^ in the dopaminergic (n = 4, t = 3.964, *p* = 0.007), serotonergic (n = 4, t = 4.554, *p* = 0.004), and octopaminergic (n = 4, t = 5.549, *p* = 0.001) neurons compared to the control flies (Figure 1).

### 3.2 Knockdown of the vesicular monoamine transporter gene affects *drosomycin* expression

Aminergic neurotransmission requires the activity of the vesicular monoamine transporter (VMAT) which packages monoamine transmitters into secretory vesicles. VMAT1 and VMAT2 are the two vesicular monoamine transporters found in mammals (Eiden et al., 2004). *Drosophila* has a single *VMAT* gene (*dVMAT*) that encodes two splice variants, dVMAT-A and -B, which diverge at their C-termini (Greer et al., 2005). *dVMAT-A* is expressed in all dopaminergic, serotonergic, and octopaminergic neurons of larvae and adults (Greer et al., 2005; Chang et al., 2006). In contrast, *dVMAT-B* is not expressed in the neurons and plays a functional role in the glia in regulating histamine in the fly’s visual system (Romero-Calderon et al., 2008). *dVMAT* mutants display various behavioral deficits in agreement with the loss of exocytotic amine release in the nervous system (Simon et al., 2009).

**FIGURE 2:**
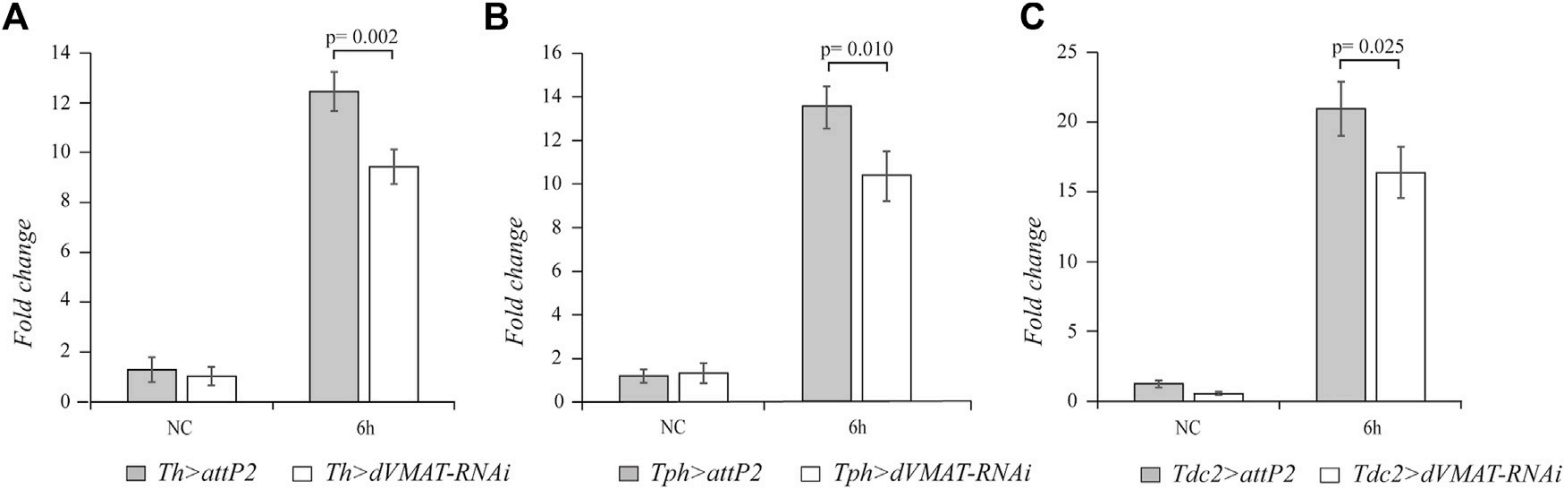
The knockdown of *dVMAT* in aminergic neurons impacts *drs* expression. qRT-PCR analysis of *drs* transcript levels at 6 h after *Micrococcus luteus* infection of adult flies, in which RNAi-mediated knockdown of d*VMAT* was induced in dopaminergic **(A)**, serotonergic **(B)**, and octopaminergic neurons **(C)** compared to their controls. NC, not challenged.

To further corroborate the finding that the neurotransmission of dopamine, serotonin, and octopamine plays a role in the systemic innate immune response of adult flies, we knocked down RNAi *dVMAT* gene. Using the *Th*-*GAL4*, *Tph*-*GAL4*, and *Tdc2*-*GAL4* drivers, we specifically expressed *dVMAT*-*RNAi* in the different aminergic neurons. The strength of the *dVMAT*-*RNAi* was checked by RT-qPCR, which showed that the *dVMAT* transcript level was strongly reduced in flies expressing *dVMAT*-*RNAi* in the nervous system using the *elav*-*GAL4* driver (*elav > dVMAT-RNAi*). (Supplementary Figure S1) (n = 4, t = 6.665, *p* = 0.001). Knockdown of *dVMAT* in the dopaminergic, serotonergic, and octopaminergic neurons caused a decrease in *drs* transcript (n = 4, t = 5.046, *p* = 0.002; n = 4, t = 3.674, *p* = 0.010; and n = 4, t = 2.955, *p* = 0.025, respectively), further supporting the positive effect of the biogenic amines on *drs* expression (Figure 2).

### 3.3 Halted dopamine, serotonin, or octopamine signaling does not affect the expression of *attacin* genes

**FIGURE 3:**
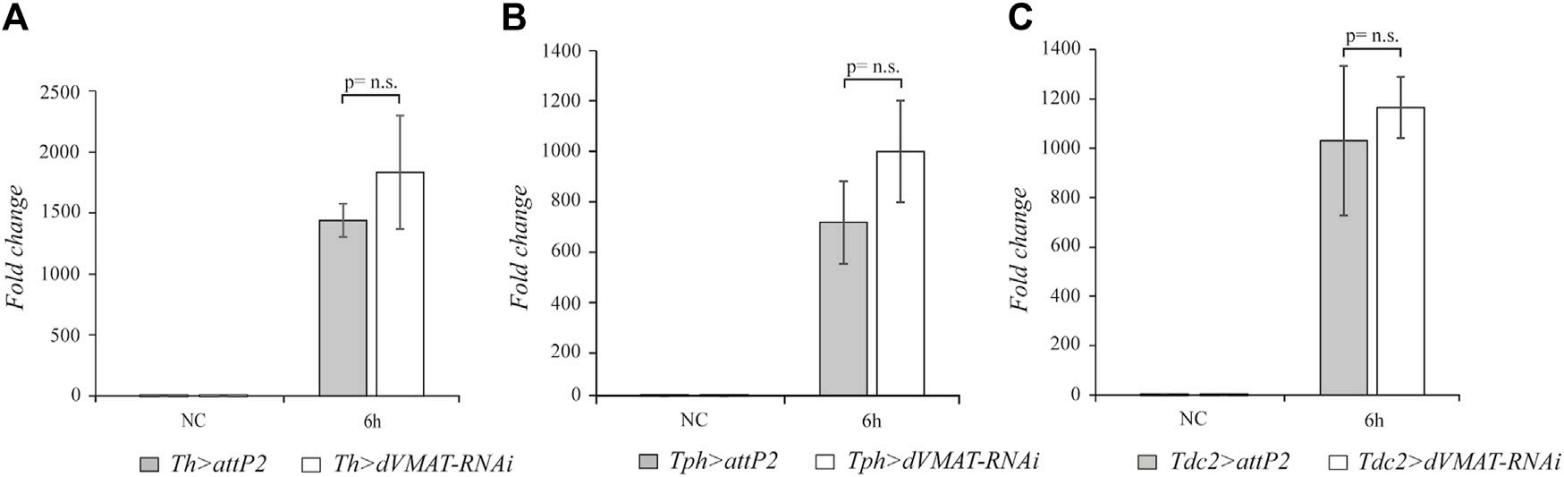
The knockdown of the aminergic signaling does not affect *att* expression. qRT-PCR analysis of *att* transcript levels at 6 h after *Micrococcus luteus* infection of adult flies, in which RNAi-mediated knockdown of *dVMAT* was induced in dopaminergic **(A)**, serotonergic **(B)**, and octopaminergic neurons **(C)** compared to their controls. NC, not challenged. Not significant (n.s.) = p≥ 0.05.

Having observed that the biogenic amine signaling positively modulates the systemic activation of *drs* expression after *M. luteus* infection, we wondered whether the aminergic signaling could target other AMP genes. The Toll pathway is the most important pathway in fighting Gram-positive bacteria; moreover, the Imd pathway, which responds mainly to Gram-negative bacteria, can also play a significant role after *M. luteus* septic injury. Indeed, double mutant flies for the Toll and Imd pathways are very sensitive to *M. luteus* infection (De Gregorio et al., 2002). We decided to analyze the effect of blocking aminergic signaling after *M*. *luteus* infection on the expression of AMP genes which appear to be under the control of both pathways. We selected the AMP *attacin* genes as the target of our experiments. The Attacins (Atts) are encoded by four genes, *att*-*A*, -*B*, -*C*, and -*D*, and their expression levels have been widely used as readouts for the Imd pathway. The att-A and att-B genes both respond to the Toll and Imd pathways (Hanson and Lemaitre, 2020), and in double mutant flies, the Toll and Imd pathways are not activated upon *M. luteus* infection (De Gregorio et al., 2002). Therefore, we checked the expression levels of these *att* genes after *dVMAT*-*RNAi* knockdown in the aminergic neurons. In our RT-qPCR analysis, we designed DNA primers to amplify both the *att*-*A* and the *att*-*B* RNAs*.* The block of aminergic signaling by *dVMAT*-*RNAi* in dopaminergic, serotonergic, and octopaminergic neurons did not statistically affect the level of *att* gene expression (n = 4, t = 1.413, *p* = 0.2074; n = 4, t = 1.889, *p* = 0.1078; and n = 4, t = 0.7157, *p* = 0.5011, respectively) (Figure 3). This suggests that by blocking aminergic signaling, while the Toll pathway is downregulated, the Imd pathway is not affected and allows the proper expression of *att* genes*.*

## 4 Discussion

The immune responses must be rapid and sufficient to control foreign invaders, but they should not be overabundant because they would be detrimental to the organism itself. The nervous system plays a central role in the modulation of immune systems. Although this is clearly demonstrated in humans, it has been less explored in insects. Our recent finding that the neurotransmitter acetylcholine plays a role in *Drosophila* immunity in the neural and non-neural cells (Giordani et al., 2023) supports that a cholinergic cross talk between the nervous and immune systems could also occur in the insect as shown for humans (Andersson and Tracey, 2012).

In the present work, we extended our analysis and investigated whether the dopamine, serotonin, and octopamine neurotransmitters, which in insects had been mainly correlated with behavioral aspects, could play a functional role in humoral immunity. We show that, after *M. luteus* infection, the induction of the proper *drs* expression level is activated by aminergic signaling. Since *drs* is mostly regulated by the Toll pathway after Gram-positive bacteria injury, we propose that aminergic signaling will end upon activating this pathway. Furthermore, by knocking down aminergic signaling, the expression of *att* genes that respond to both the Toll and Imd pathways appeared unaffected. This suggests that the Imd pathway is not targeted by aminergic signaling that could sustain the normal expression of *att* genes. However, this latest finding can be part of the complex molecular mechanisms underlying the humoral innate response. Among them, it has been reported that the Toll and Imd pathways can act synergistically in the activation of AMP genes (Tanji et al., 2007), and the impact of each pathway on the expression of each AMP gene depends on the type of infection (Leulier et al., 2000; De Gregorio et al., 2002).

In mammals, the role of aminergic signaling in innate and adaptive immunity has been clearly shown, and thus we analyzed the dopamine and serotonin functions in *Drosophila* innate immunity. In humans, dopaminergic transmission modulates the key immune cells such as neutrophils, monocytes/macrophages, NK cells, and dendritic cells (Pinoli et al., 2017). Serotonin activates monocytes and lymphocytes, thus triggering the secretion of cytokines (Herr et al., 2017). Interestingly, it has been shown that serotonin, like in human macrophages, is expressed in hemocytes and modulates insect hemocytes. These experiments were carried out in the butterfly (*Pieris rapae*) and *Drosophila* and have shown that blocking serotonin synthesis impaired hemocyte phagocytosis (Qi et al., 2016). The other amine analyzed in our work is octopamine, which is synthesized from tyramine. These amines are structurally related to the vertebrate noradrenaline and adrenaline and share an analogous physiological role. Here, we show that blocking *Drosophila* octopaminergic signaling, as it occurs for dopaminergic and serotonergic signaling, causes a reduction in *drs* expression. Nonetheless, it has been recently shown in *Caenorhabditis elegans* that octopamine is downregulated after infection to enhance immunity (Sellegounder et al., 2018). This is an interesting finding, emphasizing the complexity of immunity and its molecular mechanisms. Compared to *Drosophila*, this different strategy on octopamine activity may reflect that this nematode lacks several components of innate immunity, which are relevant parts of the immune response of *Drosophila* and other invertebrate and vertebrate organisms. The missing items include the most known pattern recognition receptors (PRRs) and NF-κB homologs (Liu and Sun, 2021).

Biogenic amines are also involved in controlling many behavioral aspects, such as olfactory learning, courtship, locomotor activity, and sleep. Sleep is essential for the development and survival of organisms, and its regulation by environmental factors such as temperature (Fan et al., 2022) and light (Mazzotta et al., 2020) is well documented in *Drosophila*. Neurotransmitters play a key role in regulating *Drosophila* sleep, with serotonin playing a sleep-promoting function opposite to the wake-promoting activity of dopamine (Ly et al., 2018). The octopamine’s role as a wake-promoting neurotransmitter is still controversial (Deng et al., 2019). Recent works in *Drosophila* highlighted the existence of different clusters of dopaminergic neurons in the mushroom bodies involved in controlling sleep and food-seeking behavior in response to a range of nutritive landscapes (Landayan et al., 2018; Tsao et al., 2018). Moreover, in *Drosophila*, as in other insects like bees (Eban-Rothschild and Bloch, 2015), the involvement of the dopaminergic system in regulating sleep in stressful social interactions is clearly established (Ganguly-Fitzgerald et al., 2006; Lone et al., 2016). More recently, it has been reported that alterations in the biogenic amines underlie depression-like behavior in *Drosophila* (Araujo et al., 2018; Hermanns et al., 2022), similar to what was observed in mammalian models (Becker et al., 2021).

Antimicrobial peptides represent an important arm of innate immunity and are produced across invertebrates, vertebrates, and plant species (Brogden, 2005). However, recent results indicate that the AMP function is not only limited to killing microbes but also involved in many other biological aspects, such as gut homeostasis, tumor control, and neurology (Hanson and Lemaitre, 2020). Studies related to neurodegenerative diseases have also implicated AMPs as contributing agents. Recent sources of evidence indicate the role of the Alzheimer’s amyloid-ß peptide as an antimicrobial peptide (Gosztyla et al., 2018) and that Alzheimer’s disease may in part be an immune process (Dominy et al., 2019). A further link between innate immune mechanisms and neurodegenerative diseases comes from the finding of an antimicrobial role of the Parkinson’s disease α-synuclein protein (Park et al., 2016). Overexpression of various AMPs using the *Drosophila* dopaminergic *Th-GAL4* driver causes an age-dependent loss of dopaminergic neurons (Shukla et al., 2019). An important question of whether the chronic activation of the immune response is just a marker of aging or whether immune effectors such as AMPs may exert a cytotoxic effect accelerating the aging-associated syndromes arises. However, it has been shown that the ubiquitous or gut-specific overexpression of AMPs significantly prolongs the lifespan of adult flies (Loch et al., 2017). Recently, Hanson and Lemaitre (2023), by analyzing the effect of an isogenic set of AMP gene deletions, found that AMPs do not directly contribute to aging in *Drosophila* but improve lifespan by preventing dysbiosis during aging.

Considering the impact of AMPs on both immunity and many other biological processes related to neurodegeneration and aging, it is of particular relevance to understand the role of the nervous system in regulating the production of these peptides using *Drosophila* as a model system. A thorough understanding of the cross-modulation between the nervous and immune systems in insects, besides its relevance from a scientific point of view, is particularly important for analyzing the impact of neurotoxic compounds, like many insecticides, on the immune systems of both target and non-target species.

## Data Availability

The raw data supporting the conclusion of this article will be made available by the authors, without undue reservation.
