# Supplementary material for: Effect of aminergic signaling on the humoral innate immunity response of Drosophila
Source: Front Physiol. 2023 Aug 24;14:1249205. doi: 10.3389/fphys.2023.1249205 (PMC10483126; doi:10.3389/fphys.2023.1249205)
Supplement: Supplementary file 1 [file Image1.pdf]

## *Supplementary Material*

# Effect of aminergic signaling on the humoral innate immunity response of *Drosophila*

**Giulia Cattabriga, Giorgia Giordani, Giuseppe Gargiulo*, Valeria Cavaliere***

*** Correspondence:**

Giuseppe Gargiulo: giuseppe.gargiulo@unibo.it

Valeria Cavaliere: valeria.cavaliere@unibo.it

## 1 Supplementary Figures

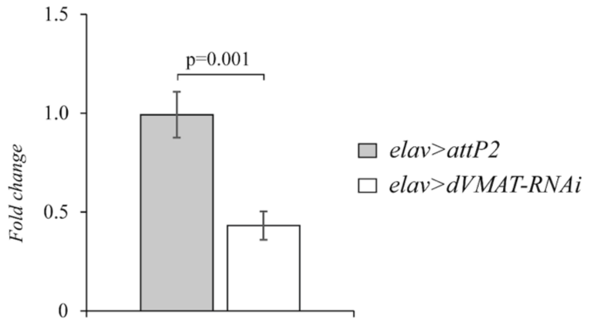


**Supplementary Figure S1.** | Gene silencing efficiency of *dVMAT-RNAi* transgene. RT-qPCR analysis of *dVMAT* transcript level in flies expressing *dVMAT-RNAi* in the nervous system using the elav-Gal4 driver (*elav>dVMAT-RNAi*) compared to the controls (*elav>attP2*). Transcript levels were normalized to *Rpl32*. Data were normalized to the control and represent the mean ± SD of 4 biological replicates, each consisting of a pool of 5 flies. Statistical significance was assessed by two-tailed unpaired Student's t- test and the relative p value reported for significant differences.
